# Different Effects of Six Antibiotics and Ten Traditional Chinese Medicines on Shiga Toxin Expression by *Escherichia coli* O157:H7

**DOI:** 10.1155/2013/121407

**Published:** 2013-07-16

**Authors:** Mei Ling Chen, Zhao Hao, Yuan Tian, Qi Yao Zhang, Pei Ji Gao, Jian Ling Jin

**Affiliations:** State Key Laboratory of Microbial Technology, School of Life Sciences, Shandong University, 27 Shanda Nanlu, Jinan 250100, China

## Abstract

This study compared the effects of ten types of traditional Chinese medicines (TCMs) and six different antibiotics on *E. coli* O157:H7 Shiga toxin gene (*stx*2) mRNA expression level based on real-time PCR and the expression level of Stx toxin using an ELISA quantitative assay. We also compared their effects on the induction of the SOS response. The results clearly indicated that all ten TCMs had negative results in the SOS response induction test, while most TCMs did not increase the levels of *stx2* mRNA and the Stx toxin. Some TCMs did increase the mRNA levels of the *stx2* gene and the Stx toxin level, but their increases were much lower than those caused by antibiotics. With the exception of cefotaxime, the six antibiotics increased the Stx toxin level and increased the *stx2* gene mRNA level. With the exceptions of cefotaxime and tetracycline, the antibiotics increased the SOS induction response. These results suggest that TCMs may have advantages compared with antibiotics, when treating *E. coli* O157:H7; TCMs did not greatly increase Stx toxin production and release.

## 1. Introduction

The major pathogen, enterohemorrhagic *Escherichia coli* (EHEC), has caused several outbreaks in different areas throughout the world, such as *E. coli* O157:H7 epidemics in many countries [1–3] and highly pathogenic *E. coli* O104:H4 epidemics in Germany and other European countries during 2011 [4, 5]. EHEC possesses multiple virulence factors and the most toxic is Shiga toxin (Stx), especially Stx2 [6–8]. EHEC can cause the life-threatening hemolytic uremic syndrome (HUS) and hemorrhagic colitis (HC) [6, 9, 10].

EHEC patients are treated mainly with supportive therapy. The use of antibiotics is not recommended, because many reports have shown that antibiotics can stimulate *E. coli* O157:H7 or *E. coli* O104:H4 to generate or release Stx, which increases the risks of HC patients becoming HUS patients [11–15]. By contrast, it also has been reported that antibiotics do not increase the expression [16–22] of the Shiga toxin-coding gene (*stx2*) so they can be used for the treatment of EHEC patients. 

For a long time, traditional Chinese medicine (TCM) has been used to treat infectious diarrhea, although people at that time did not differentiate between bacterial, nonbacterial, toxic bacterial, or nontoxic bacterial diarrhea [23, 24]. TCMs are still in use today, and they play important roles in treating infectious diseases other than enteric diseases because they are chemically complex, widely applied, not easily resisted by bacteria [25–29], and have lower toxicity [30–36]. In recent years, a hot research topic has been the extraction of effective compounds or compound complexes from TCMs to treat EHEC infections without inducing Shiga toxin overexpression. We selected ten TCMs from about fifty TCMs which are widely used in clinical Chinese medicines in China, because these TCMs had higher *E. coli* O157:H7 biofilm forming inhibition activity (data not shown in this paper). This study compared six antibiotics and these ten TCMs to assess their different effects on *stx*2 expression and the SOS response induction.

## 2. Materials and Methods

### 2.1. Bacterial Strain

Enterohemorrhagic *E. coli* O157:H7 EDL933 was kindly provided by China Disease Prevention and Control Center.

### 2.2. Antibiotics

Streptomycin was purchased from Sangon Biotech Co. Ltd, Shanghai, China (CAS no. 3810-74-0); tetracycline was purchased from Xin Jing Ke Biotechnology Co., Ltd., Beijing, China (CAS no. 3963-45-9); chloramphenicol was purchased from Guo Chang Sheng Biotechnology Co., Ltd., Beijing, China (CAS no.56-75-7); erythromycin was purchased from Bio Basic Inc., Canada (CAS no. 114-07-8); cefotaxime sodium was purchased from Qilu Pharmaceutical Co., Ltd., Jinan, China (CAS no. 64485-93-4); and hydrochloric acid levofloxacin (injection) was purchased from Yangtze River Pharmaceutical Group Co., Ltd., Yangzhou, China (CAS no. 82419-36-1). All antibiotics were stored as 50 mg/mL stock solutions at –20°C.

### 2.3. TCMs

Ten TCMs, *Coptidis Rhizoma* (CR), *Fraxini Cortex* (FC), *Schisandrae Chinensis Fructus* (SCF), *Scutellariae Radix* (SR), *Aucklandiae Radix* (AR), *Rehmanniae Radix* (RR), *Radix* et *Rhizome Rhei* (RRR), *Achyranthis Bidentatae Radix* (ABR), *Corni Fructus* (CF), *Rhizoma seu Radix Notopterygii* (RsRN), were all purchased from Beijing Tongrentang Co. Ltd., Jinan branch (Jinan, China). Their decoctions were prepared using the traditional boiling method [25, 36]. 

### 2.4. Measuring the MICs of Antibiotics and TCMs

The minimum inhibitory concentrations (MICs) of six antibiotics to *E. coli* O157:H7 EDL933 were determined with broth double dilution method [37, 38]. The MICs of ten TCMs to *E. coli* O157:H7 EDL933 were determined with agar double dilution method, because the decoctions of TCMs were somewhat turbid. 

### 2.5. Extracting RNA and Reverse Transcription *E. coli*

O157:H7 EDL933 was cultured overnight in Luria-Bertani (LB) broth. About 5 × 10^5^ colony forming units (CFUs) were mixed with serial dilutions of the antibiotics or TCMs in LB broth, followed by culture at 37°C for about 6 h with rotary shaking at 160 rpm. The bacteria cells were collected, and the total RNA was extracted strictly using a kit (Promega SV Total RNA Isolation System, Z3100), according to the manufacturer's instruction. The purity and concentration of RNA were assessed by denature agarose electrophoresis and Nano Drop. The extracted RNA samples were stored at –70°C until use. The 20 *μ*L RT-PCR reaction mixture contained 4 *μ*L 5× reaction buffer, 1 *μ*L RiboLock RNase inhibitor (20 U/*μ*L), 2 *μ*L 10 mM dNTP MIX, 1 *μ*L of RevertAid M-MuLV reverse transcriptase (200 U/*μ*L), 500 ng of total RNA, 1 *μ*L of random primers, and RNase-free H_2_O to make up the final volume to 20 *μ*L (Fermentas). The reaction was carried out at 25°C for 5 min, 42°C for 60 min, and 70°C for 5 min. The amplified cDNA samples were stored at –70°C until use. 

### 2.6. Real-Time PCR Primer Design and Reaction

The real-time PCR primers were designed according to published sequences of the EDL933 genome [39] using primer 5.0 and were synthesized by Takara Biotechnology Co., Ltd (Dalian, China). The length of the amplified *Stx2* fragment was 150 bp. The probe was 5′-(FAM) CACCGATGTGGTCCCCTGAG (Eclipse)-3′, the forward primer was 5′-CTTCGGTATCCTATTCCC-3′, and the reverse primer was 5′-GGGTGTGGTTAATAACAG-3′. *rpo*B was used as an internal control, and the length of the amplified *rpo*B fragment was 79 bp, using the probe 5′-(FAM) AACTGCCTGCGACCATCATTCT (Eclipse)-3′, the forward primer 5′-CAACCTGTTCGTACGTATC-3′, and the reverse primer 5′-CTCTGTGGTGTAGTTCAG-3′. The 20 *μ*L PCR reaction mixture contained 10.0 *μ*L of 2× Premix Ex Taq (Probe qPCR), 0.4 *μ*L of PCR forward primer (20 *μ*M), 0.4 *μ*L of PCR reverse primer (20 *μ*M), 0.8 *μ*L of fluorescent probe solution, 2.0 *μ*L of cDNA, and 6.4 *μ*L of ddH_2_O. The PCR reaction conditions were: 95°C for 15 min, 40 cycles of 95°C for 5 s, 55°C for 30 s, and 72°C for 30 s. The Ct value of each sample was the average of the real-time PCR data for triplicate samples.

### 2.7. Quantitative Determination of Stx

The amount of Stx toxin was determined using a double antibody (sandwich) ELISA with a shiga-like toxin (SLT) ELISA kit (Shanghai Jianglai Biotechnology Co., Ltd., China). Absorbance measurements were performed bichromatically at 450/600 nm with an ELISA reader. To determine the specific Stx concentration, the absolute absorbance values were divided by the number of bacteria (OD_600_ per mL) present in the suspensions.

### 2.8. The Inductive Effect of Antibiotics and TCMs on SOS Response

Using the methods recommended by ISO [40, 41], we determined the inductive effects of the antibiotics and TCMs on the SOS response at 1/8, 1/4, and 1/2 MIC concentrations, respectively.

### 2.9. Data Processing and Analysis

The 2^−ΔΔCt^ method was used for relative quantification of the real-time PCR data [42]. The statistical analyses were carried out using SPSS 13.0.

## 3. Results

### 3.1. MICs of Antibiotics and TCMs in *E. coli* O157:H7 EDL933

Table 1 shows the results obtained using the broth and agar double-dilution method to measure the MICs of antibiotics and TCMs in *E. coli* EDL933. The six antibiotics had different antibacterial mechanisms and/or different active targets, and they had much higher bacteriostatic activities than TCMs. Of the ten TCMs *Coptidis Rhizoma*, *Fraxini Cortex*, and *Schisandrae Chinensis Fructus* had high bacteriostatic activity; *Scutellariae Radix*, *Aucklandiae Radix*, and *Rehmanniae Radix* had medium bacteriostatic activity; while the other four TCMs had weak bacteriostatic activity.

### 3.2. Reproducibility and Stability of the *stx*2 Gene Expression Quantitative Measurements

**Figure 1:**
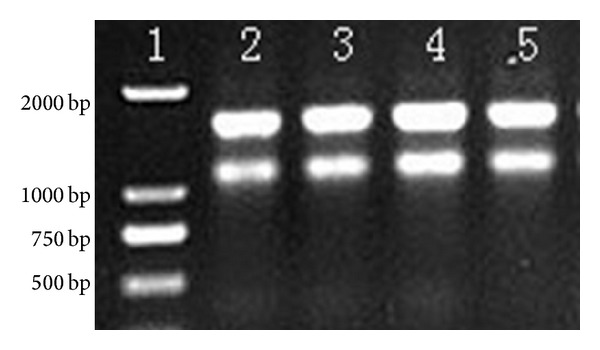
Total RNA agarose electrophoresis. 1: Trans2 K DNA marker; 2: EDL933; 3: EDL933 treated with 1/2 MIC levofloxacin; 4: EDL933 treated with 1/4 MIC levofloxacin; 5: EDL933 treated with 1/8 MIC levofloxacin.

Real-time RT-PCR was used to quantitatively compare the effects of antibiotics and TCMs on *stx*2 gene expression. We optimized the steps of real-time RT-PCR so that it had good reproducibility and stability. The optimization results indicate that the extracted RNA was of good quality and the total RNA extracted was high purity (see Figure 1), that is, A260/A280 = 2.006 ± 0.012 (*n* = 12), which indicated that there was no contamination with DNA or protein. The brightness ratio of the 23S rRNA band relative to the 16S rRNA band was about 2 : 1, so the extracted RNA was mostly complete. The real-time PCR expansion curve was generated automatically using a Roche 480 system. Fluorescent signals were not measured in the negative control group, which showed that the reaction system was free from contaminations. The same templates had similar expansion curves, which indicated that this determination method had good reproducibility, where the deviation was small and the data were credible. The expansion efficiencies of the housekeeping gene (*rpo*B) and target gene (*stx*2) were very similar, with a relative deviation of less than 5%. Thus, these relative quantitative analysis methods (2^−ΔΔCt^ method) were suitable for analyzing the effects of the antibiotics and TCMs on *stx*2 gene expression. 

### 3.3. Effects of Antibiotics and TCMs on *stx*2 Gene Expression

**Figure 2:**
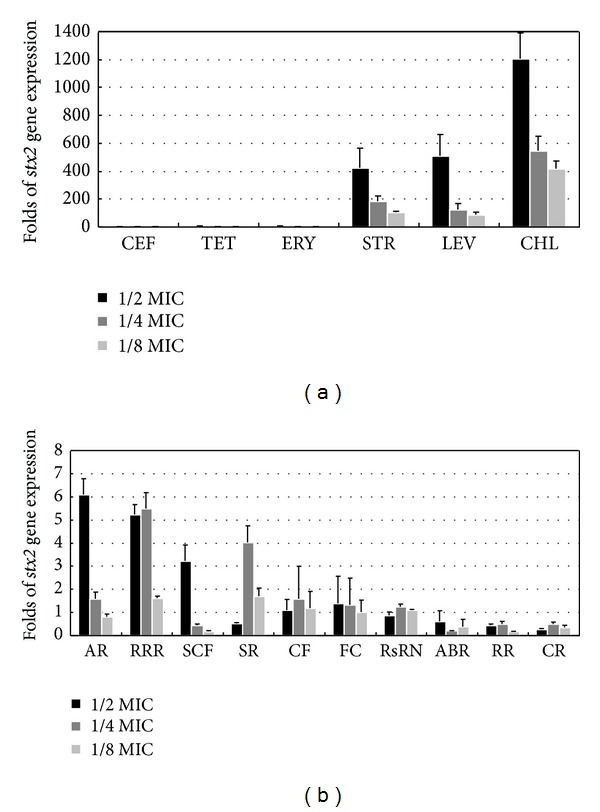
Folds increased in *stx*2 gene expression after treatments with antibiotics (a) and TCMs (b).

The effects of each antibiotic and TCM on *Stx*2 gene expression were determined at three concentrations, that is, 1/2, 1/4, and 1/8 of the MICs, respectively. Based on their expansion curves, the Ct values were calculated using the 2^−ΔΔCt^ method. The effects of the antibiotics and TCMs on *stx*2 gene expression are shown in Figure 2.

The results showed in Figure 2 indicated that chloramphenicol, levofloxacin, and streptomycin strongly increased *stx*2 gene expression in *E. coli* O157:H7 EDL933, where the maximum expression was a thousand times higher than that of the housekeeping gene. A higher antibiotic concentration correlated with greater *stx*2 gene expression. Chloramphenicol had the strongest capacity for inducting increased *stx*2 gene expression, followed by levofloxacin and streptomycin. Tetracyclines and erythromycin only showed weak induction at concentrations of 1/2 MIC. Cefotaxime sodium did not induce the expression of the *stx*2 gene at any of the three concentrations. 

The previous results also indicated that, compared with the antibiotics, six of the TCMs (CF, FC, RsRN, ABR, RR, and CR) had no significant inductive effects on *stx*2 expression, while four TCMs (AR, RRR, SCF, and SR) had weak inductive effects on *stx*2 expression at high concentrations, which were similar to tetracycline and erythromycin. Their *stx*2 expression levels were up to six times that of the control group, which were hundreds or thousands of times below that of chloramphenicol, levofloxacin, and streptomycin. CR, ABR, and RR weakly suppressed the reverse transcription and expression of *stx*2.

### 3.4. The Effects of Antibiotics and TCMs on Stx Toxin

**Figure 3:**
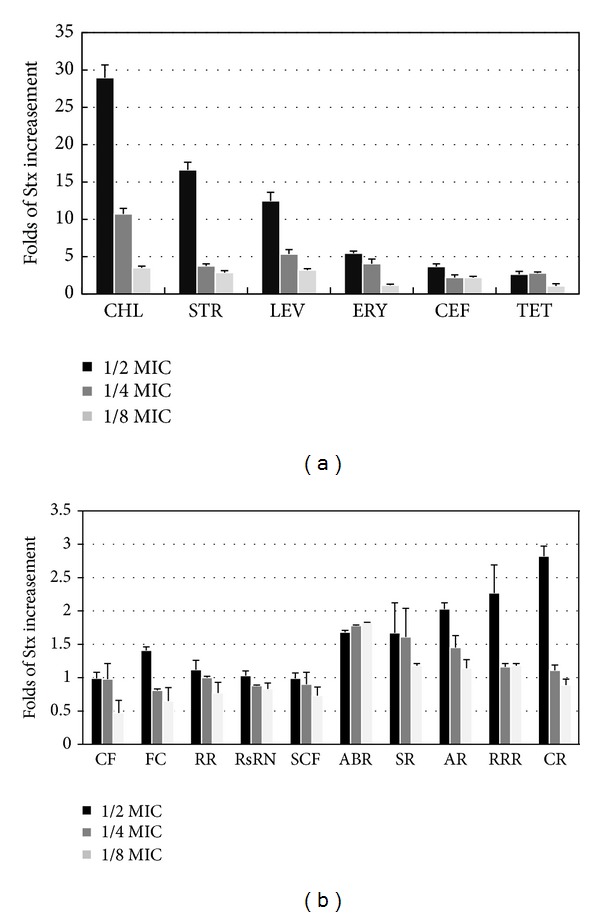
The effects of antibiotics (a) and TCMs (b) on Stx toxin.

We analyzed the Stx toxin released into the culture supernatant, and the results were shown in Figure 3. The level of Stx toxin released reflected the toxin expression level in the bacterial cells and the capacity for toxin release, including damage to cell walls and cell membranes. Thus, the Stx toxin released could reflect the effects of drugs better than the intracellular Stx toxin level. The standard curve for the quantitative ELISA analysis of Stx toxin was *Y* = 0.074*X*, *R* ^2^ = 0.990. Here, *Y* was the value of OD_450 nm_, and *X* was the amount of Stx toxin in the supernatant of the culture (pg/mL). The standard curve indicated that this method had a good linear relationship, which could be applied to the quantitative detection of Stx toxin released into the *E. coli* O157:H7 cultures after treatments with antibiotics and TCMs.

The results in Figure 3 indicated that three antibiotics (CHL, STR, and LEV) significantly increased the release of Stx toxin by over ten-fold, while three antibiotics (ERY, CEF, and TET) only weakly increased the release of Stx toxin by about 2.8–5.5 times. However, only three TCMs (AR, RRR, and CR) weakly increased the release of Stx toxin by about 2.0–2.8 times, whereas the other seven TCMs (SR, SCF, CF, FC, RsRN, ABR, and RR) did not increase the release of Stx toxin, that is, Stx toxin release increased less than two-fold.

### 3.5. Inductive Effects of Antibiotics and TCMs on SOS Response Induction

**Figure 4:**
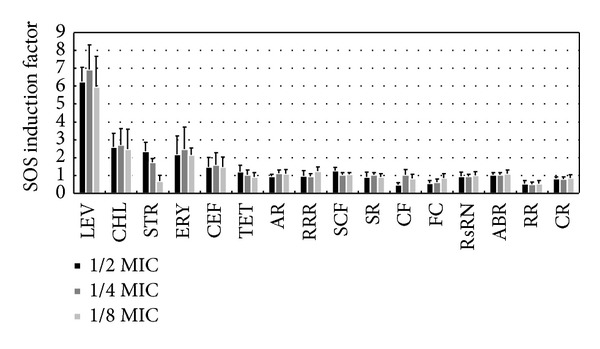
Inductive effects of antibiotics and TCMs on the SOS response. **The number of replicate experiments was ≥5 for antibiotics and ≥3 for TCMs. Each experimental trial was conducted in triplicate.

Stx toxin expression in *E. coli* O157:H7 is believed to be related to SOS response induction [43]. We also compared the different effects of antibiotics and TCMs on the SOS response induction, and the results are shown in Figure 4. The results indicated that only four antibiotics (LEV, CHL, STR, and ERY) induced a clear SOS response, and their SOS induction factors were >2.0. The other two antibiotics and all ten TCMs did not induce the SOS response, and their SOS induction factors were <2.0.

## 4. Discussion

### 4.1. The Different Inductive Effects of Six Antibiotics on *stx*2 Gene Expression in *E. coli* O157:H7

The results in Figure 2 showed that, compared with the expression of the housekeeping gene *rpo*B: chloramphenicol treatment caused a sharp increase in *stx*2 gene expression, which was thousands of times greater than the control; levofloxacin and streptomycin treatment caused increases that were hundreds of times greater than the control; the erythromycin and tetracycline treatment responses were only several times greater than the control; whereas cefotaxime did not increase *stx*2 gene expression. The fold increases of *stx*2 gene expression varied greatly among the antibiotics, but were these data distorted? First, according to the study by Ichinohe et al. [21], norfloxacin can increase the expression of *E. coli* O157:H7 *stx*2 by thousands of times compared with the control, which indicates that our data is credible. Second, our data on the effects of the six antibiotics on *stx*2 gene expression were mostly consistent with other reports, although some were contradictory. According to McGannon et al. [16], various antibiotics had different effects on the expression of *stx* gene. Antibiotics such as ciprofloxacin and sulfamethoxazole, which target the DNA, can increase *stx* gene expression greatly. However, antibiotics that target the cell wall, transcription, and translation do not increase *stx* gene expression. Interestingly, azithromycin reduces the *stx* gene expression. This hypothesis may shed some light on the data in Figure 2, such as why cefotaxime had no inductive effect, whereas tetracycline and erythromycin had relatively weak inductive effects, and levofloxacin had a strong inductive effect. The data in Figure 2 also showed that chloramphenicol and streptomycin, which affect translation, had very strong inductive effects, which contradicts McGannon et al.

There are two contrasting views of antibiotics that affect bacterial cell wall biosynthesis. A previous study [16] suggested that they do not induce *stx* gene expression, whereas another study [20] showed that ceftazidime did not affect *stx* gene expression whereas panipenem (PAPM) greatly suppressed *stx* gene expression. Other studies [17, 19] have shown that cefotaxime and meropenem [44] do not affect *stx* gene expression. By contrast, it was reported [17, 18] that ampicillin increased *stx* gene expression. However, the present study showed that cefotaxime had little inductive effect on *stx*2 gene expression.

There are also two contradictory views of antibiotics that affect biosynthesis during DNA replication. Studies have shown [14, 22, 44] that ciprofloxacin can increase *stx* gene expression and that [21] norfloxacin can increase *stx*2 expression by thousands of times compared with the control. However, another study [17] reported that ciprofloxacin did not increase *stx* gene expression, while in [12] enrofloxacin reduced *stx* gene expression. It was reported in [20] that oral intake of quinolones by *E. coli* O157:H7-infected patients did not increase the possibility of progression to HUS. The present study showed that levofloxacin strongly increased the *stx*2 gene expression, which agrees with most previous studies.

There are two contradictory views of antibiotics that affect the biosynthesis of proteins, such as aminoglycosides. One study [16] suggested that their restricted translation would not affect *stx* gene expression. Similarly, another study [44] reported that gentamicin and kanamycin did not affect *stx* gene expression, which was consistent with a previous study [26]. Another study [18] reported that gentamicin at the concentration of MIC increased the *stx*2 expression, whereas a sub-MIC concentration reduced the expression of *stx*2. However, the present study showed that streptomycin at a sub-MIC concentration (1/2, ¼, and 1/8 MIC) markedly increased *stx*2 expression.

Antibiotics that affect the biosynthesis of proteins, such as polycyclics, have been shown [16] to have no effects on *stx*2 gene expression. However, it was reported [44] that tigecyline reduced *stx*2 expression, while another study [19] reported that bicozamycin reduced *stx*2 expression, which disagreed with other work [16]. The data in Figure 2 shows that tetracycline had only a weak inductive effect on *stx*2 gene expression, which agreed with a previous report [16].

For macrolide antibiotics, such as erythromycin and its derivatives, it was reported [21] that azithromycin did not affect *stx*2 expression, whereas other studies [16, 17, 44] reported that azithromycin reduced *stx* expression. The present study found that erythromycin had only a weak inductive effect on *stx*2 gene expression, which agreed with some previous results.

A previous study [44] reported that chloramphenicol reduced *stx*2 gene expression. By contrast, we found that chloramphenicol strongly induced *stx*2 gene expression.

### 4.2. TCMs Had No or Weak Inductive Effects on *E. coli* O157:H7 *stx*2 Gene Expression

The inductive effects of antibiotics on *E. coli* O157:H7 *stx*2 expression have been reported in many studies, whereas the effects of TCMs have been reported rarely [45, 46]. In the same test conditions used for antibiotics, we found that some TCMs induced the expression of *stx*2 only weakly, such as *Aucklandiae Radix* (AR), *Radix* et *Rhizoma Rhei* (RRR), *Scutellariae Radix* (SR), and *Schisandrae Chinensis Fructus* (SCF), whereas some TCMs had no inductive effects on *stx*2 expression, such as *Rhizoma seu Radix Notopterygii* (RsRN), *Corni Fructus* (CF), and *Fraxini Cortex* (FC). Some TCMs actually suppressed *stx*2 expression, such as *Coptidis Rhizoma* (CR), *Rehmanniae Radix* (RR), and *Achyranthis Bidentatae* Radix (ABR). In general, a preliminary conclusion based on the above analyses of the mRNA levels of the *stx*2 gene, was that the inductive effects of TCMs were far less than those of the antibiotics. This may be worth exploring in greater depth.

### 4.3. Differences in the SOS Induction Response to TCMs and Antibiotics

According to the criteria of SOS/umu test system [40, 47], the induction factor (IF) ≥2.0 indicates that the tested compound can induce the SOS response. Results in Figure 4 showed that the ten TCMs did not induce the SOS response because their IF values were <2.0. For antibiotics, however, the results were complex: levofloxacin induced a very strong SOS response; chloramphenicol and erythromycin also induced high SOS responses, whereas cefotaxime, tetracycline, and kanamycin did not induce the SOS response.

TCMs did not induce the SOS response whereas some antibiotics induced strong SOS responses. Identifying the causes of these differences requires further study.

### 4.4. Explanation of the Different SOS Induction Responses of the Six Antibiotics

The present study showed that levofloxacin had the strongest SOS response inductive effect of the six antibiotics, chloramphenicol had the second strongest induction, cefotaxime had the weakest induction, while erythromycin, streptomycin, and tetracycline had the intermediate inductive effects (see Figure 4). The different SOS responses of these six antibiotics may probably be due to their antimicrobial mechanisms [48]. First, the antimicrobial mechanism of levofloxacin correlated with high SOS induction. Levofloxacin produces a bacteriostatic effect by interfering with the DNA helicase activity, which interrupts or hinders DNA replication, thereby producing DNA fragments or terminals with single strands. These DNA molecules are inducers of the SOS response [49–52]. Second, the antimicrobial mechanism of cefotaxime is correlated with low SOS induction. Cefotaxime achieves its bacteriostatic effect by inhibiting the biosynthesis of bacterial cell walls, rather than interfering with DNA replication or DNA damage repair, so cefotaxime did not induce the SOS response. Third, chloramphenicol, tetracycline, streptomycin, and erythromycin achieve their bacteriostatic effects by interfering with protein biosynthesis, although they have different specific targets [48]. However, it was difficult to understand why chloramphenicol had the highest SOS response inductive effects, whereas streptomycin and erythromycin had similar SOS response inductive effects, and tetracycline had no SOS response inductive effects. Chloramphenicol contains a chlorine atom, which dissociates from the chloramphenicol molecule to produce a chloride ion after the chloramphenicol is absorbed by bacteria cells. The chloride ion may combine with hydrogen peroxide and peroxidase in bacterial cells to form ternary complexes, which may produce reactive oxygen species (ROS) that have very strong oxidative activities [53–57]. The high oxidative effects of ROS may damage DNA molecules and produce single- and double-stranded DNA breaks, thereby inducing the SOS response [54, 55, 57].

### 4.5. Effects of Antibiotics and TCMs on the Stx Toxin of *E. coli* O157:H7

The effects of antibiotics and TCMs on the Stx toxin of *E. coli* O157:H7 were shown in Figure 3. Their effects on Stx toxin were similar to their effects on the of *stx*2 gene mRNA levels, except in the cases of cefotaxime and *Coptidis Rhizoma* (CR). Chloramphenicol, levofloxacin, and streptomycin had the strongest inductive effects on the Stx toxin of *E. coli* O157:H7, and these three antibiotics had the strongest inductive effects on the *stx*2 mRNA levels. Erythromycin and tetracycline had inductive effects on Stx toxin and the *stx*2 mRNA levels. By contrast, cefotaxime had inductive effects on Stx toxin but not on the *stx*2 mRNA levels. Of the TCMs, *Aucklandiae Radix* (AR) and *Radix* et *Rhizoma Rhei* (RRR) had weak inductive effects on the Stx toxin of *E. coli* O157:H7, and they also had weak inductive effects on the *stx*2 mRNA levels. *Coptidis Rhizoma* (CR) had weak inductive effects on Stx toxin but no inductive effects on the *stx*2 mRNA levels. All of the other seven TCMs had no inductive effects on Stx toxin, that is, *Scutellariae Radix* (SR), *Schisandrae Chinensis Fructus* (SCF) had weak inductive effects on the *stx*2 mRNA levels, whereas the other five TCMs had no inductive effects on the *stx*2 mRNA levels. Cefotaxime and *Coptidis Rhizoma* (CR) had the biggest differences in their Stx toxin and *stx*2 mRNA level effects, which may be because both of them increased the permeability of bacterial cells [25, 48].

## 5. Conclusions

Given the results of this study and our discussions, all ten of the TCMs had negative effects on SOS response induction, most of them did not increase the *stx*2 gene mRNA or Stx toxin levels, only a few TCMs increased the *stx*2 gene mRNA levels and Stx toxin levels, but their increases were many times lower than those with antibiotics. However, six antibiotics increased the Stx toxin level, increased the *stx*2 gene mRNA levels (except cefotaxime), and increased SOS response induction (except cefotaxime and tetracycline). Thus, TCMs may have advantages compared with antibiotics in the treatment of infections caused by O157:H7, that is, the TCMs treatment used to control *E. coli* O157:H7 infections might not increase Stx toxin production and release. TCMs have been used for a long time to treat infectious diseases of the digestive tract in China, this paper gives a case of experimental evidence and a possible explanation.

## Figures and Tables

**Table 1:** MICs of antibiotics TCMs in *E. coli* EDL933.

Antibiotics	MIC (*μ*g/mL)
Levofloxacin (LEV)	0.01
Streptomycin (STR)	8.00
Chloramphenicol (CHL)	1.00
Erythromycin (ERY)	3.13
Tetracyclines (TET)	6.25
Cefotaxime sodium (CEF)	0.25

TCMs	MIC (mg/mL)

*Coptidis Rhizoma* (CR)	3.9
*Fraxini Cortex* (FC)	3.9
*Schisandrae Chinensis Fructus* (SCF)	7.8
*Scutellariae Radix* (SR)	31.3
*Aucklandiae Radix* (AR)	62.5
*Rehmanniae Radix* (RR)	62.5
*Radix et Rhizome Rhei* (RRR)	125.0
*Achyranthis Bidentatae Radix* (ABR)	125.0
*Corni Fructus* (CF)	125.0
*Rhizoma seu Radix Notopterygii* (RsRN)	125.0
